# ABA represses TOR and root meristem activity through nuclear exit of the SnRK1 kinase

**DOI:** 10.1073/pnas.2204862119

**Published:** 2022-07-05

**Authors:** Borja Belda-Palazón, Mónica Costa, Tom Beeckman, Filip Rolland, Elena Baena-González

**Affiliations:** ^a^Instituto Gulbenkian de Ciência, GREEN-IT Bioresources for Sustainability (ITQB-NOVA), Oeiras, 2780-156 Portugal;; ^b^Department of Plant Biotechnology and Bioinformatics, Ghent University, B-9052 Ghent, Belgium;; ^c^VIB-UGent Center for Plant Systems Biology, B-9052 Ghent, Belgium;; ^d^Laboratory for Molecular Plant Biology, KU Leuven, 3001 Heverlee-Leuven, Belgium;; ^e^KU Leuven Plant Institute, KU Leuven, 3001 Heverlee-Leuven, Belgium

**Keywords:** root, growth control, abscisic acid, energy signaling, target of rapamycin

## Abstract

The phytohormone abscisic acid (ABA) promotes plant tolerance to major stresses such as drought, partly by modulating growth through poorly understood mechanisms. Here, we show that ABA-triggered repression of cell proliferation in the *Arabidopsis thaliana* root meristem relies on the swift subcellular relocalization of SNF1-RELATED KINASE 1 (SnRK1). Under favorable conditions, the SnRK1 catalytic subunit, SnRK1α1, is enriched in the nuclei of root cells, and this is accompanied by normal cell proliferation and meristem size. Depletion of two key drivers of ABA signaling, SnRK2.2 and SnRK2.3, causes constitutive cytoplasmic localization of SnRK1α1 and reduced meristem size, suggesting that, under nonstress conditions, SnRK2s promote growth by retaining SnRK1α1 in the nucleus. In response to ABA, SnRK1α1 translocates to the cytoplasm, and this is accompanied by inhibition of target of rapamycin (TOR), decreased cell proliferation, and reduced meristem size. Blocking nuclear export with leptomycin B abrogates ABA-driven SnRK1α1 relocalization to the cytoplasm and ABA-elicited inhibition of TOR. Furthermore, fusing SnRK1α1 to an SV40 nuclear localization signal leads to defective ABA-dependent TOR repression. Altogether, we demonstrate that SnRK2-dependent changes in SnRK1α1 subcellular localization are crucial for inhibiting TOR and root growth in response to ABA. Rapid relocalization of central regulators such as SnRK1 may represent a general strategy of eukaryotic organisms to respond to environmental changes.

The phytohormone abscisic acid (ABA) plays major roles in plant stress responses, signaling through a well-established pathway whose main effectors in *Arabidopsis* are SNF1-RELATED PROTEIN KINASE 2.2 (SnRK2.2), SnRK2.3, and SnRK2.6 (1). ABA promotes adaptation partly by modifying developmental programs and has a major impact on root architecture (2). Modulation of primary root (PR) and lateral root growth is exerted through interactions with other hormones, affecting cell division and elongation by mechanisms still poorly understood (2). We recently uncovered a connection between ABA and SnRK1 signaling that is crucial for shaping root architecture in a target of rapamycin (TOR)-dependent manner (3). TOR is a protein kinase complex that promotes cell proliferation, with its inactivation causing reduced root meristem size and defective PR growth (4). The SnRK1 kinase is activated when energy levels decline during stress, conferring protection partly by limiting growth (5). SnRK1 is also activated by ABA (3), enabling plants to repress growth, e.g. when water is scarce. Under favorable conditions, the main catalytic subunit SnRK1α1 is sequestered by SnRK2-containing repressor complexes, allowing TOR to be active. In response to ABA, these complexes dissociate, releasing SnRK2 and SnRK1α1, which inhibits TOR and growth (3). Consistent with this model, the *snrk2.2 snrk2.3* double mutant (*snrk2d*) shows defective PR growth under favorable conditions due to aberrant repression of TOR activity (3). This defect is fully rescued by the *snrk1α1* mutation, demonstrating its SnRK1α1 dependency (3).

To investigate how TOR and root growth are controlled by SnRK2 and SnRK1, we examined the root meristems of Col-0 control seedlings, the *snrk2d* mutant, and its cross with *snrk1α1* (*snrk2d/1α1*) (Fig. 1*A*). As previously reported (6, 7), treating roots with a growth-inhibitory concentration of ABA (5 µM) reduced the number of meristematic cells, leading to smaller meristems in Col-0. In contrast, *snrk2d* showed a reduction in meristem size and cell number already in control conditions (mock), and this was fully rescued by the *snrk1α1* mutation. Consistent with the ABA hyposensitivity of *snrk2d* and *snrk2d/1α1* plants (3), ABA treatment did not further decrease meristem size or cell number in these mutants (Fig. 1*A*). These meristem phenotypes correlate well with the PR length previously observed in these mutants and conditions (3) and suggest that reduced cell proliferation contributes to the reduced PR length of *snrk2d* in control conditions, mimicking the situation of Col-0 plants upon ABA treatment.

**Fig. 1.**
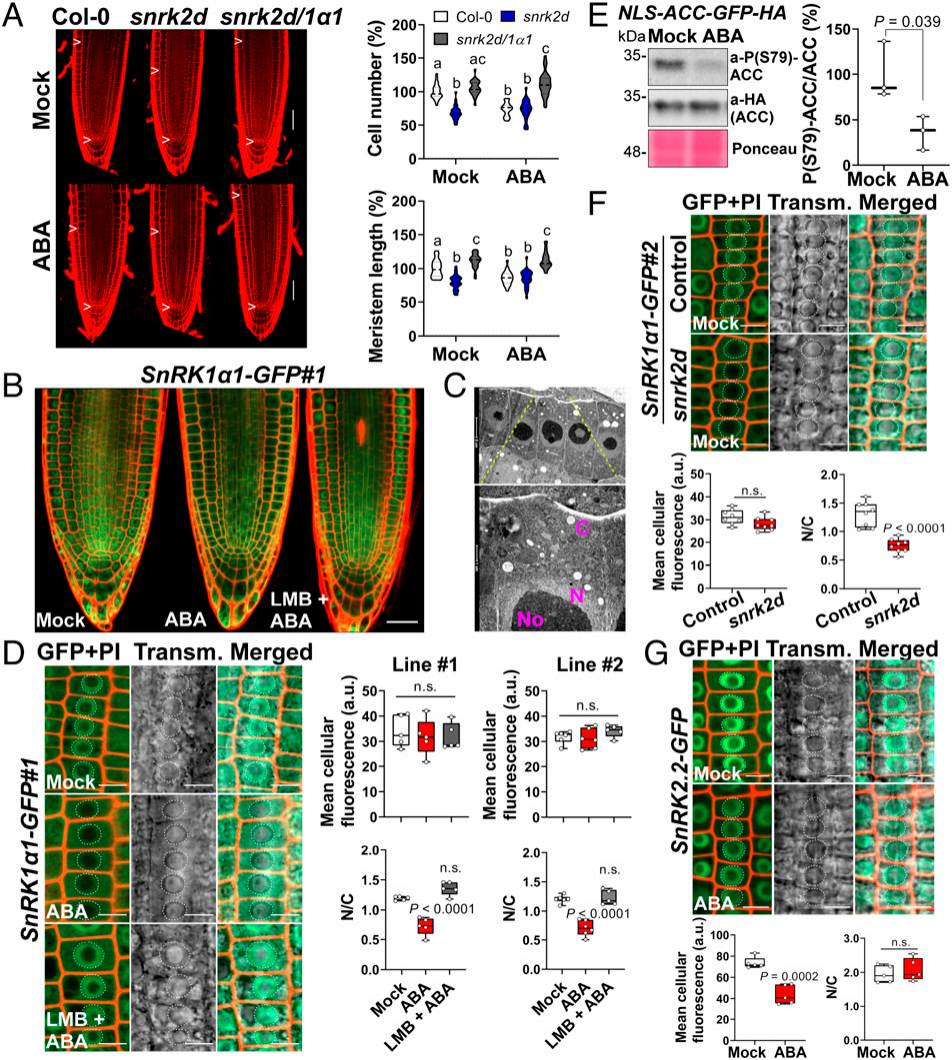
Impact of ABA and SnRK2s on SnRK1α1 subcellular localization and cell proliferation in the root apical meristem. (*A*) Meristems of Col-0, *snrk2d*, and *snrk2d/1α1* 7-d-old seedlings with or without ABA treatment (5 µM, 48 h). Arrowheads: regions used for violin plots. *n* = 23 or 24; *P* < 0.05, two-way ANOVA with Tukey’s honestly significant difference test. (Scale bar: 50 µm.) (*B*) Root apices of 4-d-old *SnRK1α1-GFP#1* seedlings with or without ABA treatment (50 µM, 3 h). (Scale bar: 30 µm.) (*C*) Electron micrograph of meristematic epidermal cells with magnification (*Lower*) showing the cellular ultrastructure. C: cytoplasm; N: nucleus; No: nucleolus. (Scale bars: *Upper*, 5 µm; *Lower*, 1 µm.) (*D*) (*Left*) SnRK1α1-GFP in epidermal cells of 4-d-old root meristems. (Scale bar: 10 μm.) Dotted lines: nuclear boundary. (*Right*) SnRK1α1-GFP quantification and N/C ratios from two independent lines. *n* = 5 or 6; one-way ANOVA with Dunett’s test. (*E*) Nuclear SnRK1 activity in 8-d-old roots of the *NLS-ACC* reporter line with or without ABA treatment (50 µM, 3 h). (*Left*) Representative immunoblot and Ponceau-S staining as loading controls. (*Right*) Quantification of ACC phosphorylation [P(S79)-ACC/total ACC]. *n* = 3, two-tailed Student *t* test. (*F*) Impact of the presence (control, *SnRK1α1-GFP#2*) or absence (*SnRK1α1-GFP#2*; *snrk2d*) of SnRK2s on SnRK1α1-GFP localization assessed as in *D*. *n* = 8; two-tailed Student *t* test. (Scale bar: 10 μm). (*G*) SnRK2.2-GFP in epidermal cells of 4-d-old root meristems treated and quantified as in *D*. *n* = 5; two-tailed Student *t* test. (Scale bar: 10 μm). PI, propidium iodide; ns, nonsignificant; a.u., arbitrary units.

We next wondered whether regulation of SnRK1 and growth by ABA and SnRK2s could involve changes in SnRK1α1 subcellular localization. First, SnRK1α1 localization is central to its function (8). Second, SnRK1 and SnRK2 are highly enriched in the nuclei of root cells (3). Third, SnRK2-harboring SnRK1 repressor complexes localize to the nucleus in *Nicotiana benthamiana* epidermal cells (3). Fourth, *in planta* the TOR complex subunit RAPTOR1B interacts with SnRK1α1 (3, 9) in the cytosol (10). We used two independent lines expressing SnRK1α1-GFP from its own regulatory regions (*SnRK1α1-GFP#1* and *SnRK1α1-GFP#2*) and imaged SnRK1α1-GFP immediately after the indicated treatments to minimize a decline of the ABA effects and potential interference from other signals (*SI Appendix*). In control conditions, SnRK1α1 displayed a known cytoplasmic and ring-shaped nuclear localization (11) (Fig. 1*B*). The latter is characteristic of nuclear proteins in meristematic cells that are absent from the nucleolus (12), whose large size is evident also in electron micrographs (Fig. 1*C*). Upon ABA treatment, the nuclear signal appeared to decline (Fig. 1*B*). Signal quantification revealed comparable SnRK1α1-GFP levels in mock- and ABA-treated roots but a reduction in the nucleus to cytoplasm ratio (N/C) from 1.19 (line #1) and 1.2 (line #2) in mock to 0.73 (line #1) and 0.71 (line #2) in ABA (Fig. 1*D*), suggesting that ABA induces SnRK1α1 nuclear exit. Accordingly, the ABA effect was fully abolished by the nuclear export inhibitor leptomycin B (LMB; N/C ratio = 1.36 and 1.23 in lines #1 and #2; Fig. 1*D*). To investigate a potential impact on SnRK1 nuclear activity, we used a line expressing a rat ACETYL-COA CARBOXYLASE (ACC) peptide, a well-established target of the SnRK1 mammalian ortholog, fused to an SV40 nuclear localization sequence (NLS) [*35S::NLS-ratACC-GFP-HA*, referred to as *NLS-ACC* (13)]. NLS-ACC protein phosphorylation therefore serves as readout of SnRK1 nuclear activity (13). Treatment of the *NLS-ACC* seedlings with ABA led to a clear reduction in ACC phosphorylation (Fig. 1*E*), indicating a decline in nuclear SnRK1 activity that is consistent with the observed nuclear exit of SnRK1α1 (Fig. 1 *B* and *D*). The rapid ABA-induced nuclear exit of SnRK1α1 and decline in nuclear SnRK1 activity are evident in other root regions and tissues(14).

Given the reduction in meristem size (Fig. 1*A*) and PR length (3) of the *snrk2d* mutant, we investigated the role of SnRK2s in SnRK1α1 localization using the cross of *snrk2d* with the *SnRK1α1-GFP#2* line (Fig. 1*F*). In mock conditions, SnRK1α1-GFP was barely present in the nuclei of *snrk2d* cells, yielding an N/C ratio of 0.75 *(*Fig. 1*F*), comparable to the ABA-treated *SnRK1α1-GFP#2* control (Fig. 1*D*). SnRK2.2-GFP underwent overall protein degradation in ABA, but its localization was not altered (Fig. 1*G*). These results show that ABA triggers nuclear exit of SnRK1α1 and that SnRK2s retain SnRK1α1 in the nucleus when ABA is not present. Intriguingly, low-energy stress enhances nuclear SnRK1 activity (ref. 13 and 14), reinforcing the view that different signals activate different SnRK1 complexes and that SnRK2s are involved in the ABA-dependent activation of SnRK1 (3).

Given that SnRK1α1 is required to inhibit TOR and growth in response to ABA (1), we investigated the role of SnRK1α1 nuclear exit in this process, using the phosphorylation of ribosomal protein S6 (RPS6^S240^) as an indirect readout of TOR activity (3). While LMB treatment had no significant impact (Fig. 2*A*, *Left*), LMB could block the repression of RPS6 phosphorylation triggered by ABA (Fig. 2*A*, *Right*). Moreover, as compared to control plants expressing wild-type SnRK1α1 (*control-α1*), the repression of TOR by ABA was also defective when SnRK1α1 was fused to an SV40 NLS that favors its presence in the nucleus [*NLS-α1* (8); Fig. 2*B*]. This demonstrates that SnRK1α1 nuclear exit is necessary for inhibiting TOR in response to ABA. Furthermore, the fact that nuclear export is crucial for repressing TOR activity (Fig. 2*A*) but only SnRK1α1 (Fig. 1*D*), and not SnRK2.2 (Fig. 1*G*), translocates to the cytoplasm in response to ABA suggests that, at least in the initial stages of the response, TOR repression is driven by SnRK1α1 and not by SnRK2.2. SnRK2s may engage in direct TOR control at later stages to reinforce the inhibition of growth (3, 15).

**Fig. 2.**
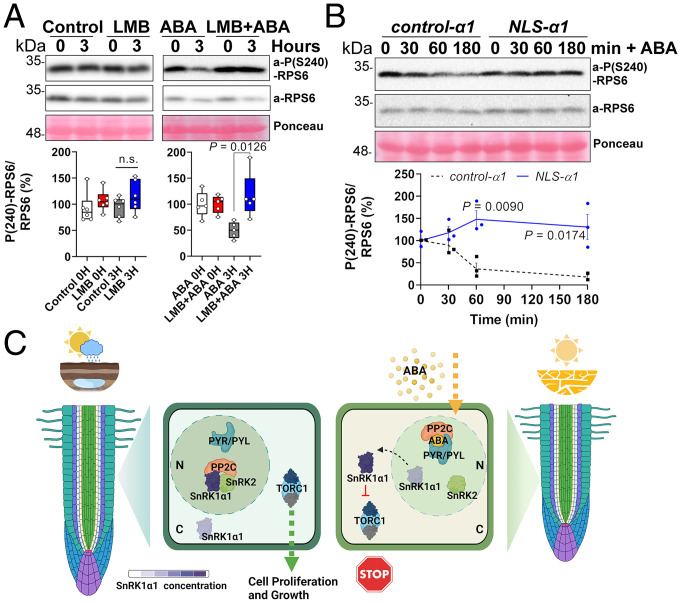
Impact of SnRK1α1 subcellular localization on TOR signaling and root growth. (*A* and *B*, *Upper*) Representative immunoblots of RPS6^S240^ phosphorylation (phospho-RPS6/total-RPS6) in Col-0 (*A*) or *control-α1* and *NLS-α1* (*B*) seedlings treated with or without ABA (50 µM, 3 h) or LMB (2.5 µM, 3 h or 1 h prior to ABA addition). Ponceau-S staining serves as loading control. Same gel images were cropped for showing *α1* and *NLS-α1* contiguously. (*A* and *B*, *Lower*) Phospho-RPS6/total-RPS6 quantification. (*A*) *n* = 5 or 6; (*B*) *n* = 3; error bars, SEM; two-tailed Student *t* test. (*C*) Under favorable conditions, SnRK1α1 is sequestered in the nucleus by complexes containing SnRK2 [and a PP2C (3)], enabling TOR activity in the cytoplasm, cell proliferation, and root growth. Dissociation of these complexes by ABA-bound PYR/PYL/RCAR receptors releases SnRK1α1, which exits the nucleus and inhibits TOR and growth. TORC1, TOR Complex 1; N, nucleus; C, cytoplasm; ns, nonsignificant. Created with BioRender.com.

We conclude that root growth is modulated by ABA through changes in SnRK1α1 subcellular localization, allowing control of TOR activity and cell proliferation in the root meristem (Fig. 2*C*). When conditions are favorable, SnRK1α1 is sequestered in the nucleus by SnRK2-containing repressor complexes. Dissociation of these complexes in response to ABA releases SnRK1α1, which exits the nucleus to inhibit TOR and growth. Mechanistic understanding of how growth is repressed by ABA may provide new means to manipulate growth-defense trade-offs in plants, enhancing stress tolerance without compromising growth and productivity.

## Materials and Methods

All experiments were done as previously described (3, 8, 13). Details are provided in *SI Appendix*.

## Supplementary Material

Supplementary File

## Data Availability

Supplementary figures have been deposited at Zenodo (https://zenodo.org/record/6566499#.Ypuhs-zMJPY) (14). All other study data are included in the article and/or *SI Appendix*.

## Change History

September 30, 2022: Figure 1 has been updated; please see accompanying Correction for details; The text of this article has been updated; please see accompanying Correction for details.
